# Potassium Channel Interacting Protein 2 (KChIP2) is not a transcriptional regulator of cardiac electrical remodeling

**DOI:** 10.1038/srep28760

**Published:** 2016-06-28

**Authors:** Sine V. Winther, Tomi Tuomainen, Rehannah Borup, Pasi Tavi, Gudrun Antoons, Morten B. Thomsen

**Affiliations:** 1 Department of Biomedical Sciences, Faculty of Health and Medical Sciences, University of Copenhagen, Copenhagen, Denmark; 2 Department of Biotechnology and Molecular Medicine, A.I. Virtanen Institute for Molecular Sciences, Faculty of Health Sciences, University of Eastern Finland, Kuopio, Finland; 3 Center for Genomic Medicine, Copenhagen University Hospital, Rigshospitalet, Copenhagen, Denmark; 4 Department of Physiology, Cardiovascular Research Institute Maastricht, Maastricht University, Maastricht, The Netherlands

## Abstract

The heart-failure relevant Potassium Channel Interacting Protein 2 (KChIP2) augments Ca_V_1.2 and K_V_4.3. KChIP3 represses Ca_V_1.2 transcription in cardiomyocytes via interaction with regulatory DNA elements. Hence, we tested nuclear presence of KChIP2 and if KChIP2 translocates into the nucleus in a Ca^2+^ dependent manner. Cardiac biopsies from human heart-failure patients and healthy donor controls showed that nuclear KChIP2 abundance was significantly increased in heart failure; however, this was secondary to a large variation of total KChIP2 content. Administration of ouabain did not increase KChIP2 content in nuclear protein fractions in anesthetized mice. KChIP2 was expressed in cell lines, and Ca^2+^ ionophores were applied in a concentration- and time-dependent manner. The cell lines had KChIP2-immunoreactive protein in the nucleus in the absence of treatments to modulate intracellular Ca^2+^ concentration. Neither increasing nor decreasing intracellular Ca^2+^ concentrations caused translocation of KChIP2. Microarray analysis did not identify relief of transcriptional repression in murine KChIP2^−/−^ heart samples. We conclude that although there is a baseline presence of KChIP2 in the nucleus both *in vivo* and *in vitro*, KChIP2 does not directly regulate transcriptional activity. Moreover, the nuclear transport of KChIP2 is not dependent on Ca^2+^. Thus, KChIP2 does not function as a conventional transcription factor in the heart.

Heart failure is a disease of the elderly^1^, and the prevalence is rising both in Western and developing countries following the general longer lifespan^2^. Heart failure is characterized by decreased pump efficiency leading to symptoms like shortness of breath, edema and fatigue. Besides the left ventricular structural remodeling seen in HF^2^, electrophysiological modifications are taking place as well. The most dominant electrophysiological changes in heart failure are modulations of repolarizing ion currents prolonging the cardiac action potential^3^,^4^,^5^,^6^ and changes in the intracellular Ca^2+^ handling^7^,^8^ aiming to improve cardiac pump function, but as a byproduct the electrical remodeling creates a substrate for ventricular arrhythmias. Underlying these structural and electrophysiological remodeling processes lie a range of transcriptional alterations where the regulating mechanisms are largely unknown^9^.

Potassium Channel Interacting Proteins (KChIPs) are accessory β-subunit proteins belonging to a family of small Ca^2+^-binding cytosolic proteins consisting of four isoforms, KChIP1-4. KChIP1-3 are structurally related as they all contain a highly conserved C-terminal core region of approximately 180 amino acids that house the 4 EF-hand domains capable of binding Ca^2+^ ions which induces a conformational change in the protein^10^. These 3 isoforms share a common mode of action on potassium channel kinetics: They all increase peak K_V_4 current, slow channel inactivation and quickens the recovery from inactivation. All isoforms are expressed in brain, but KChIP2 is the only isoform expressed in the heart, where it regulates both the repolarizing K_V_4.3 current^4^,^11^,^12^ and the depolarizing Ca_V_1.2 current^8^,^13^,^14^,^15^. The heightened interest of KChIP2 comes from its reported downregulation in HF^16^,^17^, which is thought to contribute to the modified repolarization and altered Ca^2+^ handling in the disease.

It has been shown that KChIP3 besides its electrophysiological effects also holds properties that affect gene transcription. KChIP3, primarily expressed in brain tissue^11^,^18^, has also independently been identified as Downstream Regulatory Element Agonist Modulator (DREAM)^19^ and as calsenilin^20^. KChIP3 translocates from the cytosol to the nucleus upon a raise in cytosolic Ca^2+^ concentration following activation of a calmodulin kinase II (CaMKII) mediated pathway^21^. In the nucleus, KChIP3 only binds DNA in a Ca^2+^-free state, whereas binding of Ca^2+^ to KChIP3 leads to conformational changes preventing binding to DNA^19^,^22^. KChIP3 binds Downstream Regulatory Elements (DRE) on the DNA and represses transcription of several genes, including Na^+^/Ca^2+^ exchanger 3 in cerebellar neurons^22^, prodynorphin, involved in pain sensation^19^,^23^, and Ca_V_1.2 channel in neonatal rat cardiomyocytes^21^.

It has been shown that in addition to KChIP3, the other KChIP isoforms bind DRE sites^24^, so based on this and the structural familiarity between KChIP2 and KChIP3, we investigated whether KChIP2 have transcriptional regulatory functions. If KChIP2 proves to be transcriptionally active, the repression of KChIP2-controlled genes would potentially be lost in HF as a result of the downregulation of the protein. At the same time, an increase in the intracellular Ca^2+^ concentration seen in HF would promote translocation of KChIP2 from the cytosol to the nucleus. In the present study, we investigated whether KChIP2 can translocate from the cytosol to the nucleus and tested whether KChIP2 binds DNA and alters gene expression.

## Results

### KChIP2 expression and localization in human failing and healthy hearts

**Figure 1:**
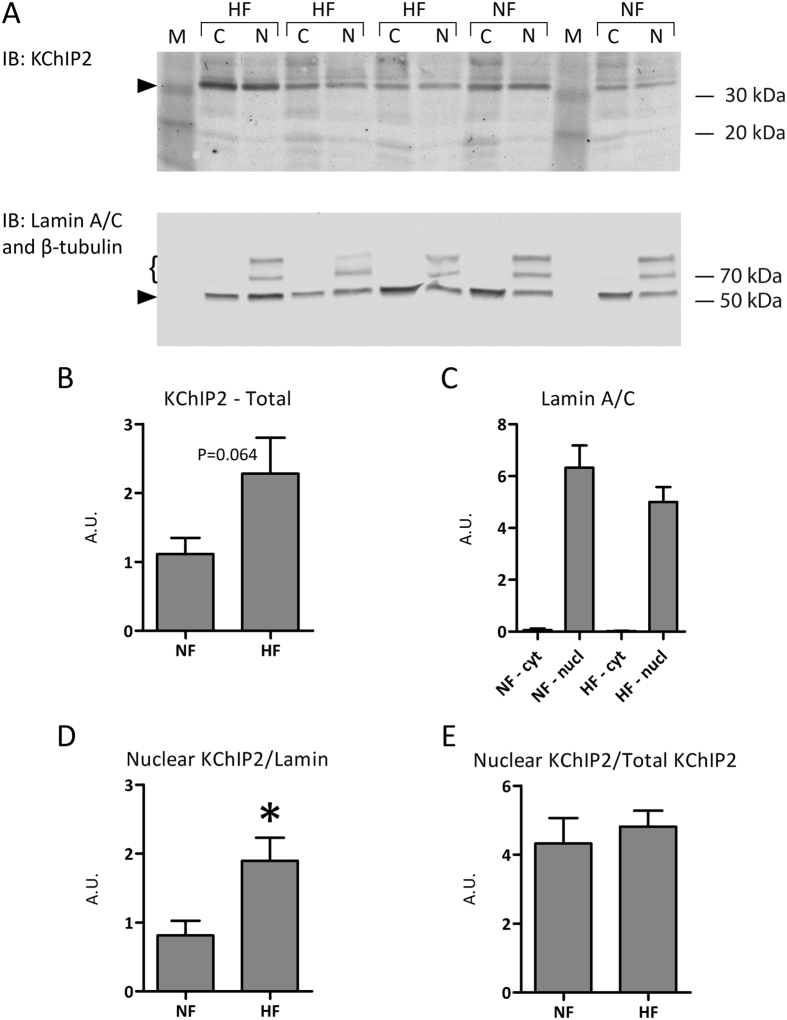
KChIP2 expression and subcellular localization in human hearts. **(A)** Representative immunoblots showing detection of KChIP2 in cytosolic (C) and nuclear (N) protein fractions from heart samples from heart-failure (HF; n = 3) and non-failing (NF; n = 2) patients. Lanes with molecular weight marker are indicated with M. Arrowheads indicate KChIP2 and β-tubulin bands in the upper and lower panel, respectively. The double band of lamin A/C is indicated with a “{”. Detecting β-tubulin and Lamin A/C confirms equal loading of cytosolic protein and successful fractionation, respectively. Each sample is analyzed in duplicate. **(B)** Quantification of the total amount of KChIP2 in the two experimental groups. **(C)** Quantification of Lamin A/C in nuclear and cytosolic fractions. **(D)** Ratio of the nuclear KChIP2 and nuclear Lamin A/C in NF and HF samples (*P = 0.025). **(E)** Ratio of the nuclear KChIP2 to total KChIP2. P values from Student’s t test.

Heart failure is a disease characterized by an elevated adrenergic state, resulting in an chronic increased intracellular Ca^2+^ concentration^25^, suggesting that the nuclear fraction of KChIP2 could be increased in this disease. Tissue samples were obtained from hearts from heart-failure patients with an ejection fraction of 22 ± 9% (n = 5) and from healthy hearts (ejection fraction 66 ± 2%; n = 5, p < 0.05; Table 1). We found that total protein levels were comparable in human samples from failing and non-failing cardiac tissue. KChIP2 migrated as a single band to the expected size around 32 kDa on immunoblots (Fig. 1A). We observed comparable total KChIP2 levels in human non-failing and failing heart samples; although there was a trend towards increased total KChIP2 levels in samples from failing hearts (P = 0.064; Fig. 1B). Successful separation of nuclear and cytosolic fractions was confirmed by the presence of Lamin A/C in nuclear fractions only (Fig. 1C). KChIP2 was present in both cytosolic and nuclear fractions, but the nuclear KChIP2 content was significantly larger in failing hearts relative to non-failing hearts (P = 0.025; Fig. 1D). Notwithstanding, the difference in nuclear KChIP2 content seemed to be dependent on the non-significant change in total KChIP2, since the nuclear-to-total KChIP2 ratio was comparable in failing and non-failing hearts (P = 0.6). Hence, in human hearts, KChIP2 is present in the nucleus; however, the nuclear-to-cytosolic ratio is not altered in end-stage heart failure.

### Nuclear translocation *in vivo*

**Figure 2:**
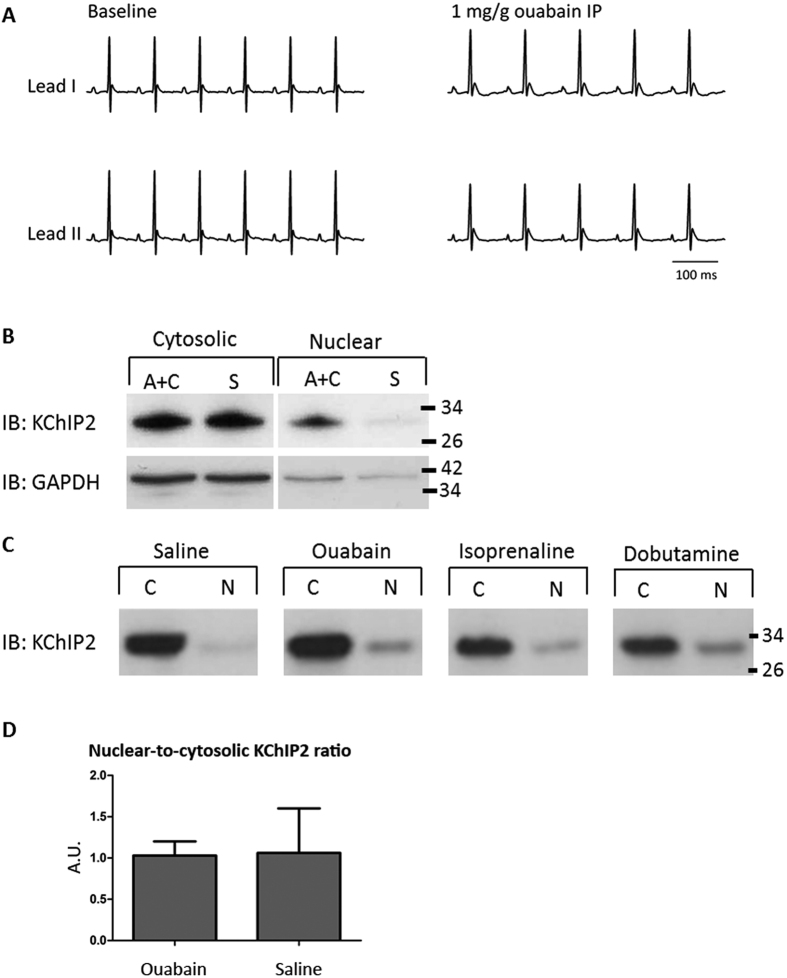
KChIP2 expression and subcellular localization in mouse hearts. **(A)** Representative two lead surface ECG from anesthetized mice at baseline and after treatment with 1 mg/g ouabain IP. **(B)** Exemplary immunoblots showing an increase of KChIP2 in the nuclear fraction when the mouse was treated with adrenaline and caffeine (A + C). Saline (S) treated mice served as controls. **(C)** Representative immunoblots showing the cytosolic (C) and nuclear (N) content of KChIP2 after administration of ouabain, isoprenaline and dobutamine, all increasing heart rate and inotropy, and saline as control. **(D)** Quantification of the nuclear-to-cytosolic ratio of KChIP2 in hearts from 5 ouabain-treated and 4 saline-treated mice and compared with Student’s t test.

Next, we investigated whether it is possible to induce a KChIP2 translocation process into the nucleus in cardiomyocytes *in vivo*. Mice were injected with a range of pharmacological compounds increasing the heart rate and/or inotropic state and thereby the intracellular Ca^2+^ concentration^26^, with the aim of initiating an acute KChIP2 translocation to the nucleus (Fig. 2A). Mice treated with adrenaline and caffeine showed an increase of nuclear KChIP2 in cardiomyocytes compared to controls in most experiments, *e.g.*, Fig. 2B; however not consistently. Ouabain, isoprenaline and dobutamine had no consistent effect on KChIP2 localization, *e.g.* the nuclear-to-cytosolic ratios of KChIP2 after administration of saline or ouabain were comparable (Fig. 2C,D). Generally, the variation between the translocation responses in the treated mice was large and the outcomes did not relate to the chronotropic or inotropic effects of the pharmacological compounds. Overall, we did not identify statistically significant KChIP2 translocation based on the pharmacologically induced acute elevation of intracellular Ca^2+^ *in vivo*.

### Nuclear translocation *in vitro*

**Figure 3:**
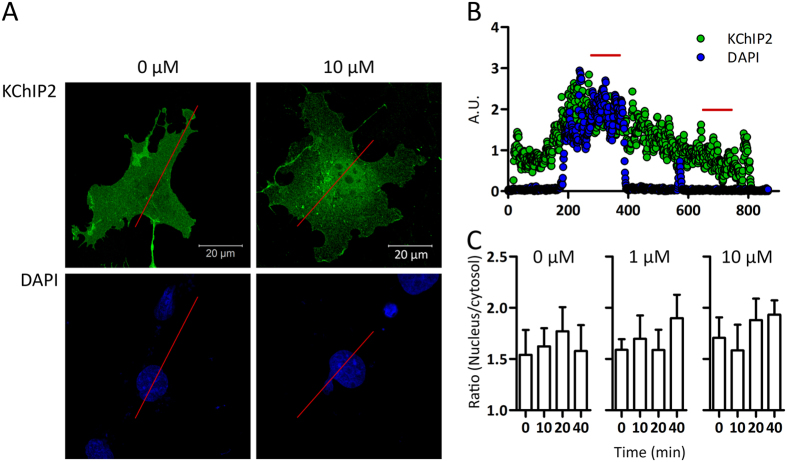
KChIP2 localization in heterologous expression systems. **(A)** COS-1 cells transfected with KChIP2 and treated with 0 μM and 10 μM ionomycin for 40 min. The extracellular [Ca^2+^] was 1.8 mM. Pictures showing representative cells stained using a KChIP2 antibody (green). The nucleus is stained blue using DAPI. **(B)** Graph showing the intensity of the KChIP2 and DAPI signals along the red line in panel A for the cell treated with 10 μM ionomycin. One hundred consecutive values were chosen within both the cytosol and nucleus for quantification of protein localization (indicated by red lines). **(C)** Quantitative analysis of the time- and concentration-dependency of KChIP2 translocation from the cytosol to the nucleus using ionomycin as a trigger. Five cells from each transfection (n = 4–5) were chosen for quantification and compared using a 1-way ANOVA.

To directly visualize the subcellular localization of KChIP2 and determining if an increase in the intracellular Ca^2+^ concentration is a triggering factor for translocation from the cytosol to the nucleus, KChIP2-transfected cells were treated in a time- and concentration dependent manner with Ca^2+^ ionophores (Fig. 3). The transfected cells, in absence of treatments to elevate the intracellular Ca^2+^ concentration, had already significant levels of KChIP2-immunoreactive protein in the nucleus (Fig. 3A). Increasing the intracellular Ca^2+^ concentration caused no change in nuclear levels of KChIP2 (Fig. 3C). Comparable results were obtained when treating the cells with another Ca^2+^ ionophore (A23187, data not shown).

**Figure 4:**
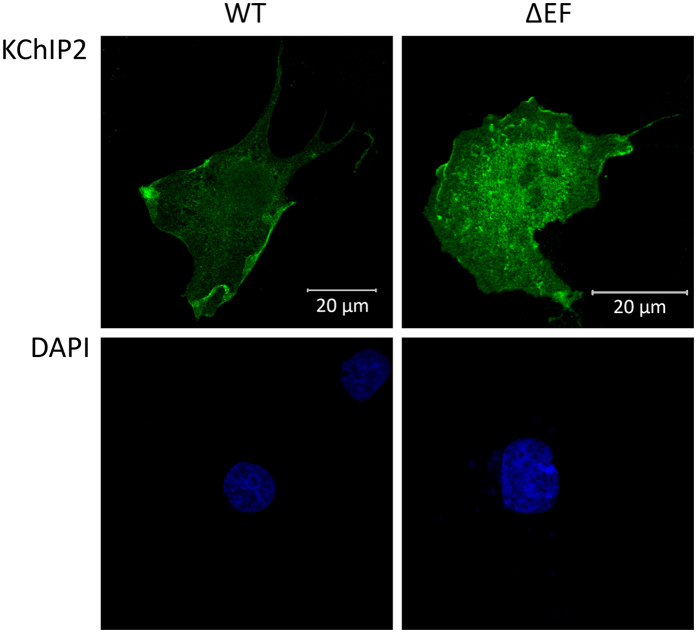
Regular KChIP2 (WT) and KChIP2-∆EF (∆EF) expression and localization in COS-1 cells. COS-1 cells transfected with WT KChIP2 and KChIP2-∆EF and treated with 10 μM ionomycin for 40 min. Pictures showing representative cells stained using a KChIP2 antibody (green). The nucleus is stained blue using DAPI. Both KChIP2 and KChIP2-∆EF are present in the nucleus.

We transfected cells with the Ca^2+^-insensitive KChIP2-∆EF mutant to determine if KChIP2’s Ca^2+^ sensitivity is responsible for translocation. There was no difference in KChIP2 localization between control cells and cells treated with Ca^2+^ ionophores in medium containing a physiological level of Ca^2+^ (Fig. 4). These experiments corroborate the *in-vivo* studies and show that KChIP2 is present in the nucleus of transfected, un-stimulated cells and they suggest that increases in intracellular Ca^2+^ concentrations do not trigger a translocation of KChIP2 from the cytosol into the nucleus in heterologous expression systems.

### Localization of KChIP2 in Ca^2+^-depleted cells

**Figure 5:**
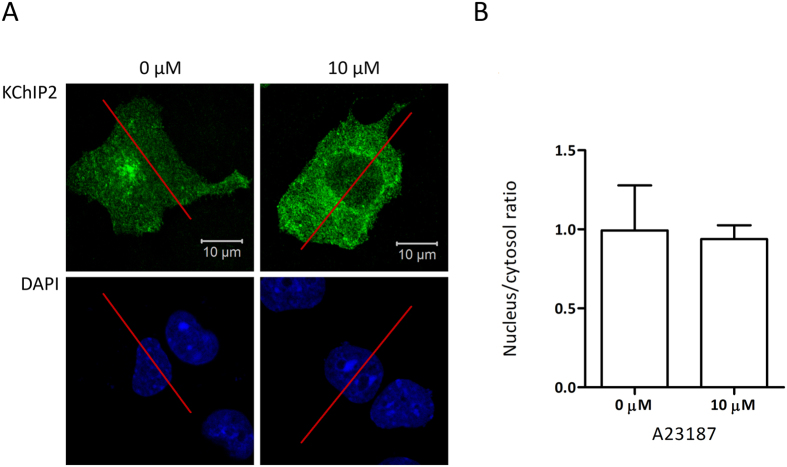
Ca^2+^ depletion in HL-1 cells. **(A)** HL-1 cells transfected with KChIP2 cDNA and treated with 0 μM A23187 in a Ca^2+^-containing medium or 10 μM A23187 in a Ca^2+^-free medium for 40 min. Pictures showing representative cells stained using a KChIP2 antibody (green). The nucleus is stained blue using DAPI. **(B)** Quantitative analysis of KChIP2 localization after Ca^2+^ depletion and the control. Five cells from each transfection (n = 2–3) were chosen for quantification and compared using a Student’s t test.

KChIP2 localization in the nucleus in absence of stimulation indicates that the Ca^2+^ concentration during normal conditions is already elevated to a level that allows translocation, or that KChIP2 is independent of a raise in the intracellular Ca^2+^ concentration in order to translocate to the nucleus. To test this, we observed protein localization in a Ca^2+^-free environment. HL-1 cells overexpressing KChIP2 were incubated for 40 min in a Ca^2+^ free medium and treated with A23187 in order to deplete intracellular Ca^2+^ (Fig. 5). The variations within both groups were large, but on average the nucleus/cytosol ratio did not differ in Ca^2+^-depleted and control cells. Thus, the presence of KChIP2 in the nucleus is not regulated by Ca^2+^.

### KChIP2 DNA binding studies using the *Cacna1c* promoter region

**Figure 6:**
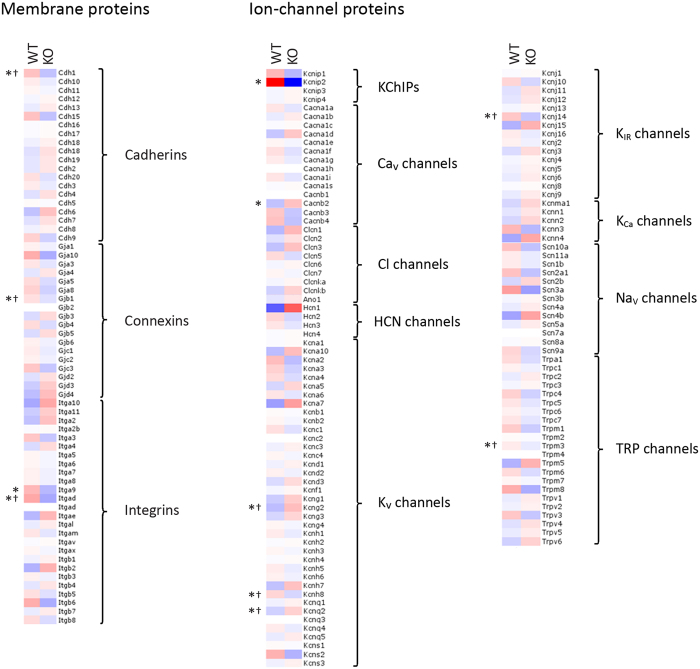
Expression of genes encoding membrane and ion-channel proteins in WT (n = 3) and KChIP2^−/−^ (n = 3) hearts using the Affymetrix Mouse Gene 482 1.0 array. Colours indicate log_2_-transformed and mean normalized expression levels from high (red) to low (blue) shown in pseudo colours of the z-score (standard deviations from the mean). *P < 0.05 (Student’s t test). ^†^Gene not expressed above the level of background noise (indicated for genes with statistically significant differences only). Integrin α-9 (Itga9) and the β_2_ subunit of the L-type Ca^2+^ channel (Cacn2b) were expressed at 82% and 135%, respectively, in KChIP2^−/−^ hearts, relative to WT. KChIP2 was not detected in KChIP2^−/−^ hearts.

To test if KChIP2 regulates gene expression in general, we performed expression studies. If KChIP2 is an important transcript factor *in vivo*, we expect that transcription of a large number of genes would be altered. Microarray data detected the expression of 14,905 genes in the WT mouse hearts; however, only 37 genes were expressed with a statistically significant difference in KChIP2^−/−^ hearts that exceeded a 1.4 fold cut-off (Table 2). Figure 6 depicts the expression levels of membrane and ion-channel proteins in WT and KChIP2^−/−^ hearts. Most changes in gene expression are very minor and involved mostly genes which were detected near background levels (log_2_ values below 5), indicating that KChIP2 is not a transcriptional modifier. Finally, the microarray confirmed that KChIP2 is the only KChIP isoform expressed in the heart, and showed that no other KChIP isoform is expressed in KChIP2^−/−^ hearts (Fig. 6).

The *Cacna1c* promoter region contains a Downstream Regulatory Element, a reported KChIP3 binding site^21^, whereby KChIP3 can repress transcriptional activity. In the microarray analysis, there was no difference in expression levels of *Cacna1c* in WT and KChIP2^−/−^ hearts. To confirm the finding that KChIP2 does now affect transcription of this gene, we used a chromatin immunoprecipitation assay on neonatal mouse cardiomyocytes treated with Ca^2+^ ionophores and used the obtained DNA as a template for quantitative PCR. Despite using 2 different KChIP2 antibodies, the amount of precipitated input DNA was low relative to control, indicating that KChIP2 does not bind to DNA under the present conditions. Thus, the present experiments show that KChIP2 does not function as a repressor of gene expression *in vivo* in the mouse and that KChIP2 does not bind DNA *in vitro*.

## Discussion

Three proteins were described practically simultaneously, and we now know that these proteins are identical: Calsenilin was initially described as a calcium-binding protein that binds presenilin, which is critically involved in early-onset familial Alzheimer disease^20^. Downstream regulatory element antagonist modulator (DREAM) was described as a calcium-regulated transcriptional repressor of key genes involved in memory and pain^19^. Finally, the potassium-channel interacting proteins (KChIP) were described as important modulators of the neuronal A-type potassium current, which is comparable to the cardiac transient outward potassium current (*I*_to_)^11^. Later, it was clear that calsenilin, DREAM and KChIP3 are the same.

In the heart, only KChIP2 is expressed. It significantly modulates *I*_to_^11^,^4^,^27^,^28^, and recently we have identified direct functional interaction between KChIP2 and the cardiac calcium channel: KChIP2 binding to the cytosolic N-terminal domain of Ca_V_1.2 causes a 40% increase in current amplitude^8^,^13^,^15^. KChIP1, 2 and 3 share 70% amino acid identity with an identical core domain containing 4 EF hands, of which 3 can bind Ca^2+^^11^. In neurons, KChIP3 is upregulated in KChIP2^−/−^ mice, and KChIP2 is upregulated in KChIP3^−/−^ mice^29^, suggesting a compensatory expression profile displayed by the two proteins and potentially a complementary function. In the present study, none of the KChIPs are expressed in hearts from KChIP2^−/−^ mice (Fig. 6).

In 2011, Ronkainen and colleagues^21^ identified a mechanism by which the calcium concentration of the cell could transcriptionally feedback on the calcium channel itself: A downstream regulatory element (DRE) in the promoter region of *Cacna1c* was sensitive to CaMKII-mediated repression of transcription. When CaMKII was activated by rising cytosolic [Ca^2+^], KChIP3 would translocate from the cytosol to the nucleus, bind to the DRE and repress transcription of *Cacna1c*, the gene encoding Ca_V_1.2, the cardiac calcium channel. Based on the overall similarity between KChIP2 and -3 and given the physiological expression of KChIP2 in the heart, we decided to test the hypothesis that KChIP2 was transcriptionally active.

KChIP2 is palmitoylated at the N-terminal which causes effective localization of KChIP2 to the cytoplasmic side of the plasma membrane and is required for achieving the full effect on *I*_to_ in heterologous expression systems^30^. Palmitoylation is the addition of palmitic acid derivatives to cysteine residues, and it has been suggested that it may not serve as a targeting signal *per se*, but rather stabilize the ion channel complex at the cell surface^30^. Both KChIP2 and -3 has a comparable cellular distribution, with clear staining in both cytoplasm and in nuclei in primary neuronal cultures^18^. In the present study, we show that KChIP2 can be identified in nuclear protein fractions from cardiac tissue from humans and from mice, and we show by immunofluorescence that cell lines transiently overexpressing KChIP2 have immunoreactive protein in the nucleus.

From the present study, it is clear that KChIP2 is present in the nucleus; however, it is unclear what function the protein has there, far from its described interaction partners at the cell surface. Small molecules and proteins up to a weight of ~40 kDa can passively diffuse through the nuclear pore complexes^31^, which could suggest that KChIP2 (32 kDa) is merely present in the nucleus as a consequence of non-regulation. Nevertheless, the documented regulation of the nuclear import of KChIP3 (28 kDa) is a clear example of a small, transcriptionally active protein that is actively transported to the nucleus when cytosolic calcium increases.

Using a host of stimulation protocols *in vivo* and *in vitro*, we were unable to unequivocally initiate a translocation process stimulating the transport of KChIP2 into the nucleus. Moreover, microarray data from KChIP2^−/−^ hearts suggested that KChIP2 was transcriptionally inactive and the ChIP assay failed to show specific KChIP2-DNA interaction. Hence, based on these experiments, we conclude that unlike KChIP3, the nuclear presence of KChIP2 is not regulated by cytosolic calcium concentrations and KChIP2 does not interact with the DRE’s to repress transcriptional activity. One important limitation of the ChIP assay is that it is very useful for detecting proteins that binds DNA; however, it is not very specific in discriminating false negative and true negative outcomes.

The data shows that KChIP2 does not directly regulate transcription; however KChIP2 augments both sarcolemmal K^+^ currents^4^,^11^,^12^ and Ca^2+^ currents^8^,^13^,^15^,^32^ with complex impact on action potential morphology^8^,^32^. This may set the stage for an electrical-transcriptional feedback loop, where current amplitudes and membrane potentials signals to the nucleus, which have been described previously, *e.g.*, during atrial arrhythmias^33^. Thus, changes in KChIP2 levels may cause cardiac remodelling via this electrical-transcriptional coupling; however, a direct effect of KChIP2 on transcription is not likely.

Soltysinska and colleagues^16^ have previously shown that KChIP2 mRNA and protein levels are significantly reduced in ventricular tissue samples from end-stage heart-failure patients (New York Heart Association class IV). In the present study, we found a surprising trend (P = 0.064) towards upregulated total KChIP2 protein in samples from failing hearts (Fig. 1C). Heart samples and sources differed in the two studies; however, the primary antibody was identical. The age of the healthy donors may have contributed to the difference in results, as our donors were older (59 years of age versus 42 years of age in the Soltysinska study) and thereby potentially had lower KChIP2 expression. It is clear that we need larger and better controlled studies of the proteomic changes that occur in the heart with disease and with age. Notwithstanding, KChIP2 may not be unequivocally downregulated in heart disease.

In conclusion, we show that KChIP2 does not translocate from the cytosol to the nucleus in a regulated manner and that KChIP2 cannot bind DNA or modulate gene expression as a conventional transcription factor. The potential function of nuclear KChIP2 is presently unknown. Moreover, our data suggest that the general assumption that KChIP2 reduction in heart failure is the primary electrophysiological hallmark of the disease may not be universal.

## Methods

### Ethical considerations and experimental animals

Human left ventricular tissue was harvested from end-stage failing hearts removed during heart transplantation or from donor hearts not suitable for transplantation as approved by the Local Ethics Committee (Ref No. 20–277 ex 08/09) of Medical University of Graz, Austria. All procedures were carried out in accordance with the approved guideline and the patients had all provided written informed consent. None of the donors had a clinical history of heart failure and were classified as non-failing with preserved ejection fraction according to echocardiographic assessment before explant.

Animal experiments were performed using male C57BL6 (wild type) and KChIP2^−/−^ mice. Mice were genotyped (GeneTyper, NY, USA) using DNA isolated from tail samples. Animals had access to water and food *ad libitum* and were housed in a room with a temperature of 22 °C and a 12 h light/dark schedule. Body temperature was kept at 37 °C during surgical procedures. Euthanasia was done by cervical dislocation at the end of the experiments. The experiments were approved by the national ethics committee (The Ministry of Food, Agriculture and Fisheries, Denmark) and were carried out in accordance with the approved guidelines. Further, the experiments conform to *The Council Directive of the European Communities on The Protection of Animals used for Scientific Purposes (2010/63/EU)* and the declaration of Helsinki.

### KChIP2 localization *in vivo*

Mice were anesthetized with 1.5% isoflurane and surface ECG and core temperature was monitored throughout the experiment. Adrenaline (2 μg/g) and caffeine (120 μg/g) was co-administered intraperitoneally (IP) in order to raise the intracellular Ca^2+^ concentration in the cardiomyocytes. In addition, dobutamine (2 μg/g IP), ouabain (1 mg/g IP or 60 μg/min intravenously (IV)) or isoprenaline (2 μg/g IP) were tested. Mice injected with saline served as control. The hearts were explanted after 10 minutes treatment and snap frozen in liquid N_2_. Tissue from human and mouse hearts were stored at −80 °C until use.

The tissues were homogenized using Precellys system (Bertin Technologies, France) and the proteins were isolated and divided into cytosolic and nuclear fractions using NE-PER fractionation kit following the manufacturer’s instructions (Thermo Scientific, MA, USA). Total protein concentration in the samples was determined using a Lowry protein assay (BioRad, CA, USA). Human and murine samples were separated on 4–15% TGX SDS-PAGE or 4–20% Tris-HCl SDS-PAGE (BioRad), transferred to immobilon hybond-p polyvinylidene flouride (PVDF) transfer membranes (Millipore, MA, USA) and blocked in Odyssey blocking buffer (LI-COR, NE, USA). The membranes were blocked in 4% milk in PBS. Primary antibody incubation was done overnight at 4 °C using a KChIP2 antibody (3.47 μg/ml, NeuroMab, UC Davis, CA, USA). Lamin A/C antibody (Cell Signaling Technology, MA, USA) was used to test if the fractionation protocol was successful, as lamin A/C is confined to the nucleus. GAPDH (0.2 μg/ml, Sigma-Aldrich, MO, USA) and β-tubulin antibodies (1 μg/ml, Millipore, MA, USA) served as loading control: Total (nuclear + cytosolic) content should be comparable between subjects; however, it is not possible to compare protein loading between cytosolic and nuclear lanes. Immunoreactive proteins were detected using fluorescent donkey anti-mouse and donkey anti-rabbit antibodies (0.1 μg/ml, LI-COR, NE, USA), or with peroxidase conjungated donkey anti-mouse and donkey anti-rabbit antibodies (0.08 μg/ml, Jackson Immunoresearch, PA, USA). Visualization of the proteins was done using the Odyssey system (LI-COR, NE, USA) and enhanced chemiluminescence for human and murine samples, respectively. Each sample was tested in duplicate. Densitometric quantification of the bands was performed with the Image Studio (LI-COR, NE, USA) and ImageJ software.

### KChIP2 translocation *in vitro*

Experiments were performed using COS-1 cells cultured in Dulbecco’s Modified Eagles Medium (DMEM) containing 10% Fetal Bovine Serum (FBS) and 1% penicillin-streptomyocin, and HL-1 cells cultured in Claycomb medium (Sigma-Aldrich, MO, USA) supplemented with 2 mM L-glutamine, 100 μM noradrenaline, 10% modified FBS and 1% penicillin-streptomyocin. COS-1 and HL-1 cells were plated in 35 mm dishes in DMEM and Claycomb medium, respectively, for 24 hours and transiently transfected using SilentFect (Invitrogen, CA, USA) according to the manufacturer’s instructions. Human KChIP2.1 in a pXOOM vector was used for transfections. In some experiments a Ca^2+^-insensitive KChIP2 mutant with asparagine-to-alanine substitutions in the Ca^2+^-binding EF hands (KChIP2-∆EF) was used to probe calcium-dependency of translocation^15^.

Transfected COS-1 cells were treated with either 0, 1 or 10 μM ionomycin for 0, 10, 20 or 40 min or with 0 or 10 μM A23187 (Sigma-Aldrich) for 0 or 40 min, in order to elevate the intracellular Ca^2+^ concentration. The Ca^2+^ ionophores were dissolved in DMSO and diluted in DMEM to reach final concentrations. Moreover, HL-1 cells were treated with 10 μM A23187 in Ca^2+^ free DMEM for 40 min. Cells treated with DMSO in Ca^2+^-containing DMEM served as control. In both experiments the cells were fixed in 4% paraformaldehyde for 20 min. KChIP2 was stained using a KChIP2 antibody (10.4 μg/ml, NeuroMab) and fluorescent secondary antibody (4 μg/ml, Alexa Flour 488 donkey anti-mouse, Invitrogen) and the nuclei were stained using 4′,6-diamidino-2-phenylindole (DAPI, 16.7 μg/ml). Protein localization was visualized using confocal microscopy (LSM710, Zeiss) and ImageJ software was used to quantify the fluorescent signal across the cell. This was done by drawing a line through both the nucleus and cytosol, using the fluorescent signal intensities as a measurement for protein density. One hundred consecutive values were chosen within both nucleus and cytosol, the values were averaged and a nucleus/cytosol ratio was calculated.

### Microarray analysis

Ventricular heart tissue from 3 wild type and 3 KChIP2^−/−^ mice was used for the analysis. The tissue was homogenized using Precellys system, and from the homogenates the RNA was isolated. NanoDrop Spectrophotometer and Flourospectrometer (both Thermo Scientific) were used for RNA concentration determination. RNA was amplified and labelled using a pico-amplification kit according to manufactures instructions. In short, 50 ng total RNA was amplified using the Ovation Pico WTA v.2 RNA Amplification System from (NuGEN, San Carlos, CA, USA) and biotin labelling was performed with the Encore Biotin Module (NuGEN). The labelled samples were hybridized to the Mouse Gene 1.0 ST GeneChip array (Affymetrix, Santa Clara, CA, USA). The arrays were washed and stained with phycoerytrin conjugated streptavidin (SAPE) using the Affymetrix Fluidics Station 450, and the arrays were scanned in the Affymetrix GeneArray 3000 7G scanner to generate fluorescent images. Cell intensity files (CEL files) were generated in the GeneChip Command Console Software (AGCC) (Affymetrix, Santa Clara, CA, USA) and imported into the software Partek Genomics Suite. The data was pre-filtered to exclude genes with an average expression level within each group (WT and KO) below noise or background level of log_2_ values of 3. Class comparison between the WT and KO was performed and a gene was defined to be differentially expressed between the two groups if p-value was below 0.05 in t-test and fold change above 1.4. For hierarchical cluster visualization of membrane proteins and ion channels, the average expression value for each of the two groups was imported into Qlucore Omics explorer v. 3.2 (Qlucore, NY, USA) and genes were normalized to have mean equal to zero.

### Chromatin ImmunoPrecipitation (ChIP)

Neonatal ventricular mouse cardiomyocytes were isolated 1–2 days postpartum and cultured as described previously^34^. Cardiomyocytes were treated with 1 μM A23187 (Sigma-Aldrich) or DMSO as control for 1 hour. Cells were fixed in 1% formaldehyde for 15 min at room temperature, washed in ice-cold PBS, scraped in Farnham lysis buffer containing Protease Inhibitor Cocktail (PIC, Roche, Switzerland), centrifuged for 5 min at 700 g and pellets were snap frozen. Pellets were then resuspended in RIPA buffer containing PIC and sonicated to an averaged chromatin size of 200–300 base pairs using Bioruptor sonicator 4 × 10 min, followed by centrifugation for 15 min at 15.000 rpm. Both monoclonal and polyclonal KChIP2 antibodies (13 μg/ml for NeuroMab and 2.5 μg/ml for Santa Cruz Biotechnology, TX, USA) were used to immunoprecipitate protein-DNA complexes. Rabbit IgG was used as negative control while KChIP3 antibodies were used for positive control. The DNA was purified using QIAquick PCR Purification Kit (Qiagen, Germany) following the manufacturer’s instructions, and the DNA fragments used as template for quantitative PCR (qPCR). KChIP2 binding was examined using primers specific for the *Cacna1c* promoter region containing a Downstream Regulatory Element (DRE) sequence, which is a putative KChIP2 binding site, or for a negative control region about 1000 base pairs downstream from the binding site.

### Statistical analysis

All data are presented as mean ± SEM. Statistical analysis were done with Student’s t-test or 1-way ANOVA for comparison of two groups or more underlying two variable conditions. P-values < 0.05 were considered statistical significant.

## Additional Information

**How to cite this article**: Winther, S. V. *et al*. Potassium Channel Interacting Protein 2 (KChIP2) is not a transcriptional regulator of cardiac electrical remodeling. *Sci. Rep.* **6**, 28760; doi: 10.1038/srep28760 (2016).

## Figures and Tables

**Table 1:** Characteristics of patients from which cardiac biopsy material were used for immunoblotting.

	Heart failure	Non-failing controls	P value
Age, years	59 ± 2	59 ± 4	0.91
Body-mass index	26 ± 1	28 ± 5	0.79
Ejection fraction, %	22 ± 9	66 ± 2	0.0003
Dilated cardiomyopathy	5/5	0/5	0.008
NYHA class III	5/5	0/5	0.008
*Medication*			
Beta-adrenergic receptor blockers	3/5	0/5	0.17
Anticoagulation	5/5	1/5	0.048
ACE-inhibitors	5/5	0/5	0.008
Digoxin	3/5	0/5	0.17
Diuretics	4/5	1/5	0.21

P values for age, body-mass index and ejection fraction derive from unpaired Student’s t-tests. Data on dilated cardiomyopathy, New York Heart Associations (NYHA) class III and medications are provided as number of patients relative to group size (5 patients in each group); these P values are from comparisons using Fisher’s exact test.

**Table 2:** Genes with significantly different and >1.4-fold changed expression in WT and KChIP2^−/−^ hearts.

Gene Symbol	Mean WT	Mean KChIP2^−/−^	P value	Ratio
*Kcnip2*	79.4	n.d.	0.00002	Only in WT
*Olfr1284*	35.4	n.d.	0.035	Only in WT
*Igfals*	38.0	n.d.	0.038	Only in WT
*Mosc1*	33.5	n.d.	0.018	Only in WT
*Cdk2ap1*	105.5	68.2	0.004	0.65
*S100a9*	63.4	41.2	0.031	0.66
*Prp2*	154.1	104.4	0.012	0.67
*Myc*	49.6	34.3	0.029	0.69
*Cyr61*	133.9	92.4	0.003	0.69
*Ormdl3*	76.6	54.0	0.003	0.70
*Dear1*	57.9	81.6	0.004	1.41
*Mrps27*	38.5	54.0	0.029	1.41
*Amd1*	242.8	341.6	0.015	1.41
*Amd1*	290.3	413.9	0.001	1.42
*Wdr23*	41.3	59.0	0.016	1.43
*Foxo3*	129.4	183.8	0.026	1.43
*Myocd*	86.2	123.4	0.001	1.43
*Hmcn1*	34.7	49.3	0.034	1.44
*Dym*	71.6	102.6	0.014	1.44
*Otub1*	84.4	122.1	0.011	1.45
*Tfb2m*	112.9	163.2	0.015	1.45
*Pik3r1*	259.6	378.1	0.045	1.46
*Plxnb2*	36.3	53.3	0.018	1.47
*Ptgds*	209.2	304.0	0.027	1.47
*Max*	232.3	337.7	0.035	1.47
*Ddit3*	86.4	127.6	0.033	1.48
*Neb*	47.7	71.9	0.042	1.49
*Gmnn*	85.2	127.4	0.001	1.49
*Amd1*	139.1	209.4	0.005	1.51
*Ephx1*	38.1	57.5	0.028	1.51
*Rnf185*	40.0	60.4	0.032	1.53
*Gdap10*	282.6	436.6	0.021	1.54
*Gpx3*	70.7	109.6	0.037	1.56
*Pih1d1*	n.d.	46.8	0.010	Only in KChIP^−/−^
*Hdhd3*	n.d.	47.2	0.039	Only in KChIP^−/−^
*Ankrd45*	n.d.	45.5	0.033	Only in KChIP^−/−^

n.d.: Not detected. P values from Student’s t tests.
